# Widely Split P Waves After Pharmacologic Cardioversion in a Patient With Atrial Fibrillation

**DOI:** 10.1016/j.jaccas.2023.102036

**Published:** 2023-09-27

**Authors:** Monika I. Shumkova, Elayne K. de Oliveira, Dario T. Bertolone, Chiara Valeriano, Isabelle Depoot, Flore Vincent, Tom De Potter, Guy Van Camp

**Affiliations:** a OLV Heart Center, Aalst, Belgium; b Department of Advanced Biomedical Sciences, University of Naples Federico II, Naples, Italy; c Department of Geriatrics, Onze Lieve Vrouwziekenhuis Hospital, Aalst, Belgium; d Department of Cardiology, Algemeen Ziekenhuis Maria Middelares, Gent, Belgium

**Keywords:** atrial tachycardia, echocardiography, electrocardiogram

## Abstract

We report a case of a clinically asymptomatic patient with extreme P-wave separation on the electrocardiogram mimicking atrial tachycardia with atrioventricular block. The accurate examination of the patient, analysis of the electrocardiogram, and echocardiographic findings led to proper diagnosis, management, and treatment. (**Level of Difficulty: Intermediate.**)

## Central Illustration

**Figure:**
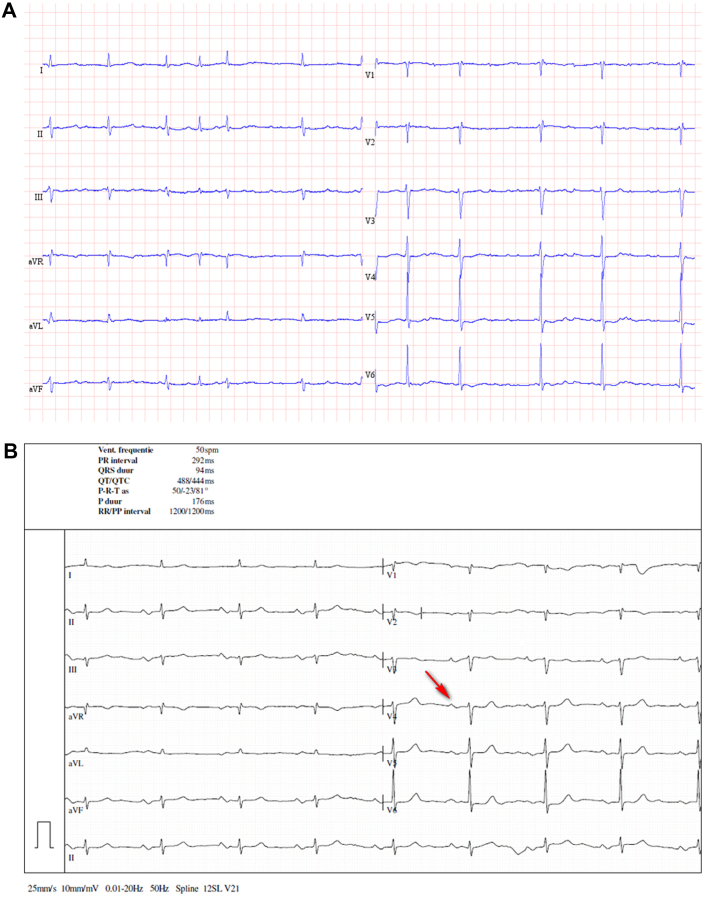

**Figure:**
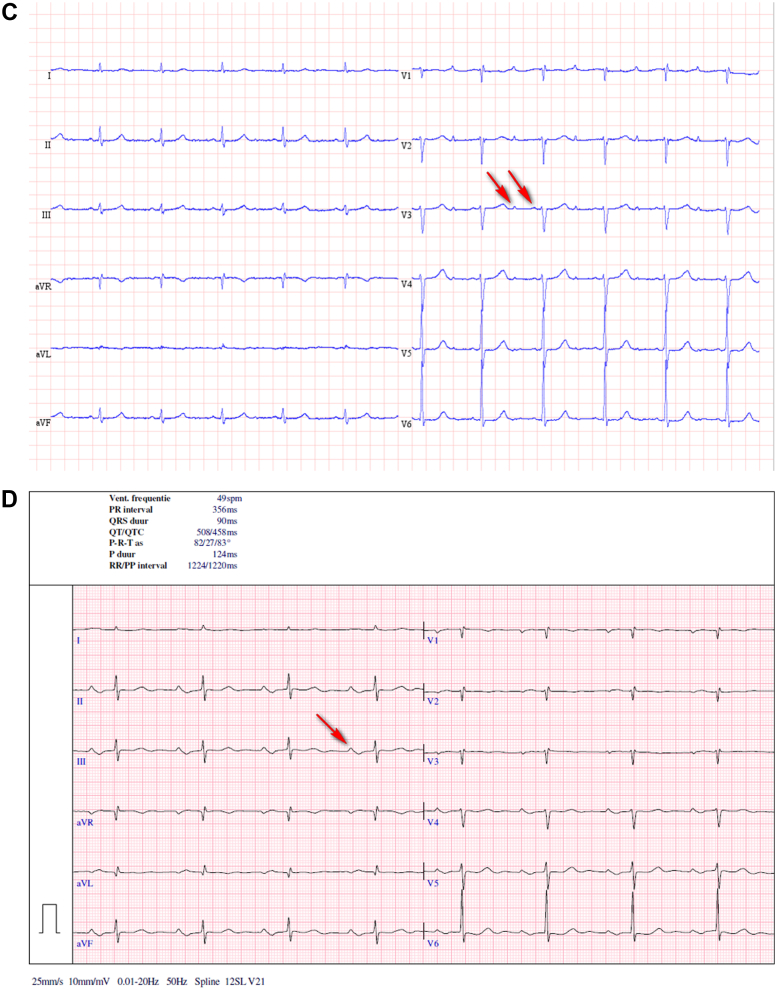

## History of Presentation

**Figure 1:**
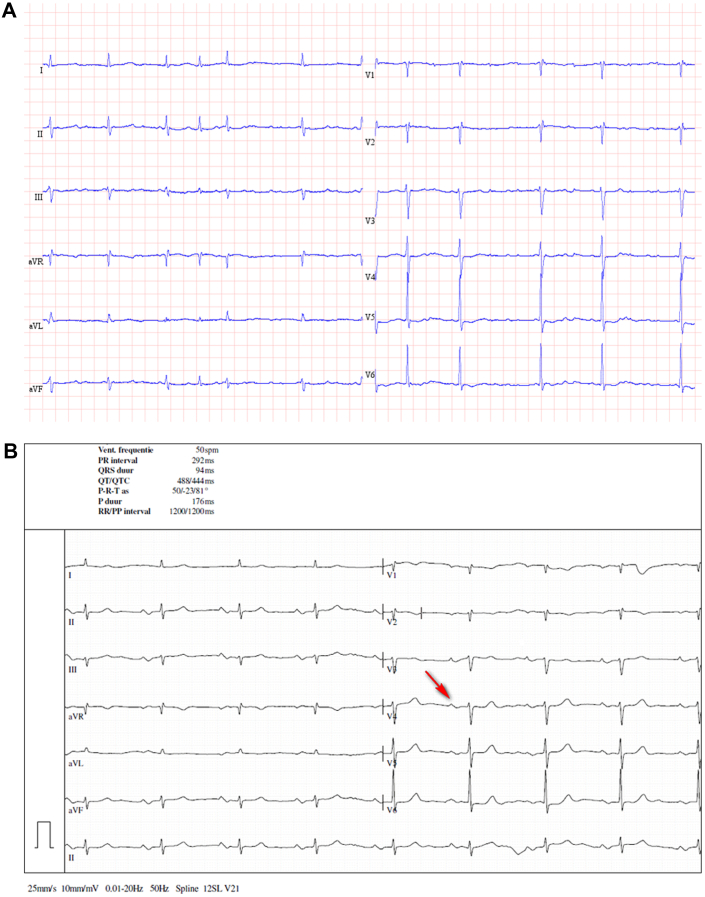

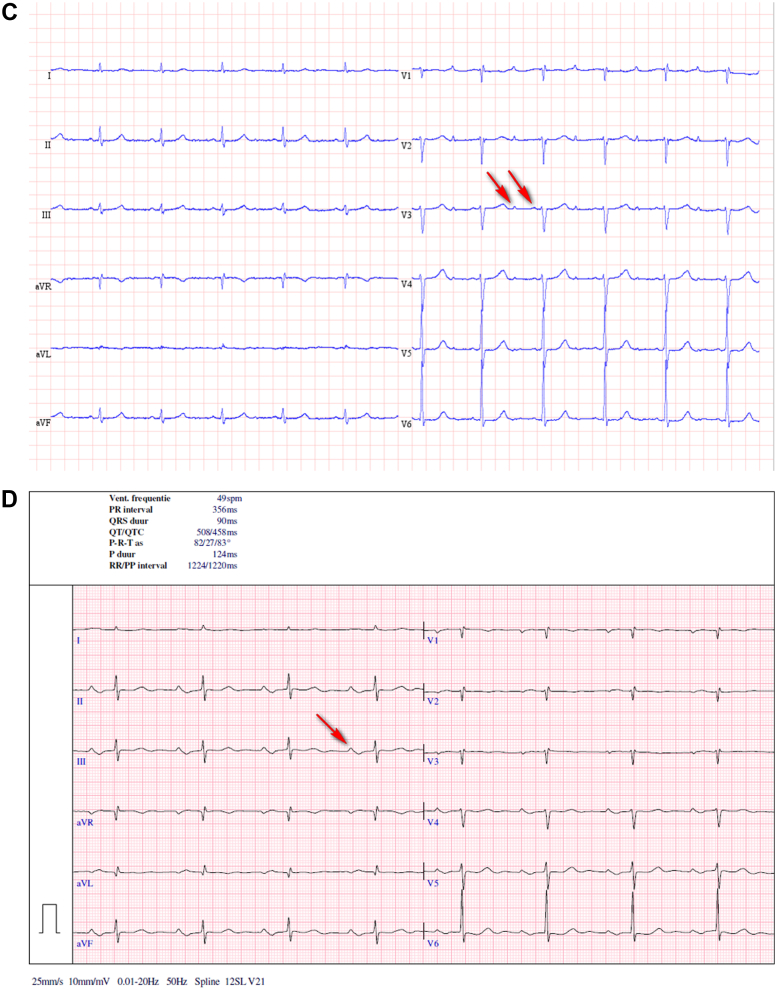
Electrocardiogram (A) Atrial fibrillation. (B) Without antiarrhythmic drugs: sinus rhythm, first-degree atrioventricular block, and biphasic (positive-negative) P waves in leads DII-DIII-aVF. (C) Therapy with flecainide: widely split P waves (red arrows) associated with each QRS complex, most distinct in leads V_3_-V_4_. Note leads DII and V_4_-V_6_ mimicking 2:1 atrial tachycardia. (D) Follow-up, without antiarrhythmic drugs: sinus rhythm and first-degree atrioventricular block. Red arrows show the P wave.

An 83-year-old woman was admitted to the emergency department with palpitations, chest pain, and shortness of breath, symptoms related to a new episode of AF (Figure 1A). Physical examination revealed blood pressure of 125/70 mm Hg, heart rate of 70 beats/min, and saturation of 94%. There were no signs of pulmonary edema. The electrocardiogram (ECG) after restoration of sinus rhythm showed widely split P waves.

**Learning Objectives**

• To be able to make a differential diagnosis between interatrial conduction delay and AT with AV block on the surface ECG.
• To emphasize the role of echocardiography in this differential diagnosis.

## Past Medical History

The patient has a medical history of paroxysmal atrial fibrillation (AF), arterial hypertension, left ventricular diastolic dysfunction, and polymyalgia rheumatica. She was on therapy with a beta blocker (amiodarone was stopped a few days before), edoxaban, and prednisolone. She underwent pulmonary vein isolation (PVI) for AF 9 years earlier. The patient is known to have a first-degree atrioventricular (AV) block and incomplete right bundle branch block (Figure 1B).

## Differential Diagnosis

Differential diagnoses of split P waves include atrial tachycardia (AT) with AV block. At first glance, the surface ECG (Figure 1C) appears to show AT because there are 2 P waves present for every QRS complex. On further inspection, though, it becomes apparent that P-P′ timing is different from P′-P timing, and thus typical AT with AV block to the ventricle can be excluded. The morphology of both P waves was also slightly different. Finally, the presenting rhythm before flecainide (Figure 1A) could also be explained by AT with variable AV conduction.

## Investigations

Laboratory tests showed elevated troponin and N-terminal pro–B-type natriuretic peptide levels with normal thyroid function. Coronary angiography ruled out coronary artery disease. The patient was hemodynamically stable without any symptoms.

An ECG (Figure 1C) after the restoration of sinus rhythm showed widely split P waves associated with each QRS complex, most distinct in chest leads V3-V4. The distance between the first P-wave to the QRS complex was 400 milliseconds and from the second (P′) to the QRS complex was 140 milliseconds, yielding an interatrial conduction time (P-P′ interval) of 210 milliseconds.

**Figure 2:**
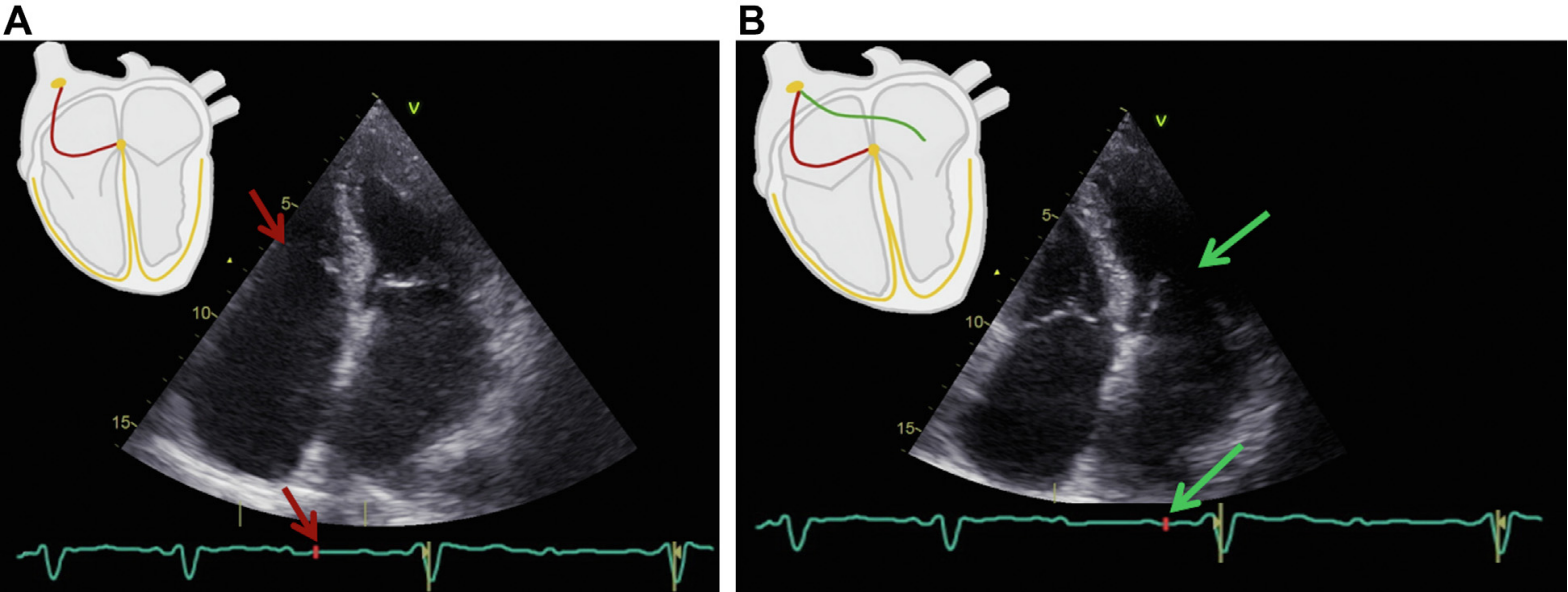
Transthoracic Echocardiography (A) Activation of the right atrium (red lines) with opening of the tricuspid valve and appearance of the first P-wave (red arrows). (B) Activation of the left atrium (green lines) with opening of the mitral valve (green arrows) and appearance of the second P-wave.

A transthoracic echocardiography revealed preserved left ventricular ejection fraction and biatrial dilatation with moderate mitral and tricuspid regurgitation. Prolonged activation time between the right atrium (RA) and left atrium (LA) is seen during the examination with a significant difference in the time between the opening of the AV valves (Video 1). Figure 2A demonstrates that the opening of the tricuspid valve (depolarization of the RA) coincides with the appearance of the first P-wave on the ECG, and in Figure 2B, the opening of the mitral valve (depolarization of the LA) occurs simultaneously with the second P-wave.

Based on the patient’s medical history, this was the first episode of AF after the PVI procedure. During these years, she presented with first-degree AV block, with an AV conduction time of 240 milliseconds and incomplete right bundle branch block.

## Management

Following admission to the cardiology department, sinus rhythm was restored using intravenous flecainide. Given the initial clinical presentation with an episode of AF, the presence of split P waves on the ECG, and the absence of clinical symptoms, the final management was to exclude the antiarrhythmic drugs with a close follow-up.

## Discussion

We report a case of a patient with prolonged interatrial activation time resulting in extremely split P waves on the surface ECG. The first clinical case was recorded in 1941.^1^ In 1997, Soejima et al^2^ presented a patient with a 10-year history of syncope with split P waves. Following an electrophysiologic study, they found a conduction disturbance in the upper and the lower RA. Chen et al^3^ described a patient with a preoperative appearance of split P waves, with a presentation of Mobitz type II during general anesthesia, treated with pacemaker implantation. In these case reports, patients are presented with symptoms requiring treatment (pacemaker implantation). Our patient was completely asymptomatic during sinus rhythm.

The appearance of the P-wave on the ECG is a marker of electrical activation of the atria. A P-wave consists of 2 parts: the first is depolarization of the RA, and the second appears after propagation of the impulse to the LA through the Bachmann bundle and reveals the activation of the LA. Structural or conduction disorders in these pathways can lead to the presence of split P waves.^4^

Although the P-wave morphology of both P waves in our case is certainly not conventional, they appear to support an activation originating in the RA first (supported by dominant amplitudes in the right precordials first) and the LA second. The activation also exhibits craniocaudal propagation as seen in the limb leads, and a posterior-to-anterior activation in the RA (the first P-wave). Although this last finding is not expected for a normal sinus rhythm, it appears consistent with RA activation in isolation. On the other hand, no invasive mapping was performed, and therefore an ectopic atrial rhythm from the RA cannot be excluded. An argument supporting an ectopic rhythm would be that an exaggeration of pre-existing conduction delay is to be expected.

The P-wave abnormalities are a result of an atrial enlargement and remodeling. Split P waves are a sign of electromechanical dysfunction. They are known as a substrate for supraventricular arrhythmias, predominantly AF.^5^ Bachmann^1^ found that that the main pathomorphologic process is the replacement of muscle tissue with fibrosis. Ischemic heart disease, PVI, and related medication use were also described as etiologic factors.

When we analyzed the ECGs of our patient, the extreme interatrial delay was seen when the patient was taking sotalol in the past, amiodarone (before hospitalization), and flecainide with a bisoprolol (during the current hospitalization). Figures 1B to 1D show the ECGs before starting the antiarrhythmic drugs; during the hospitalization, with the use of flecainide and bisoprolol and, probably, still amiodarone impregnation’ and the follow-up, without any antiarrhythmic drugs. In our patient, given the transient nature of P-wave splitting, antiarrhythmic drug–induced interatrial conduction delay is the most likely explanation for the observed findings.

**Figure 3:**
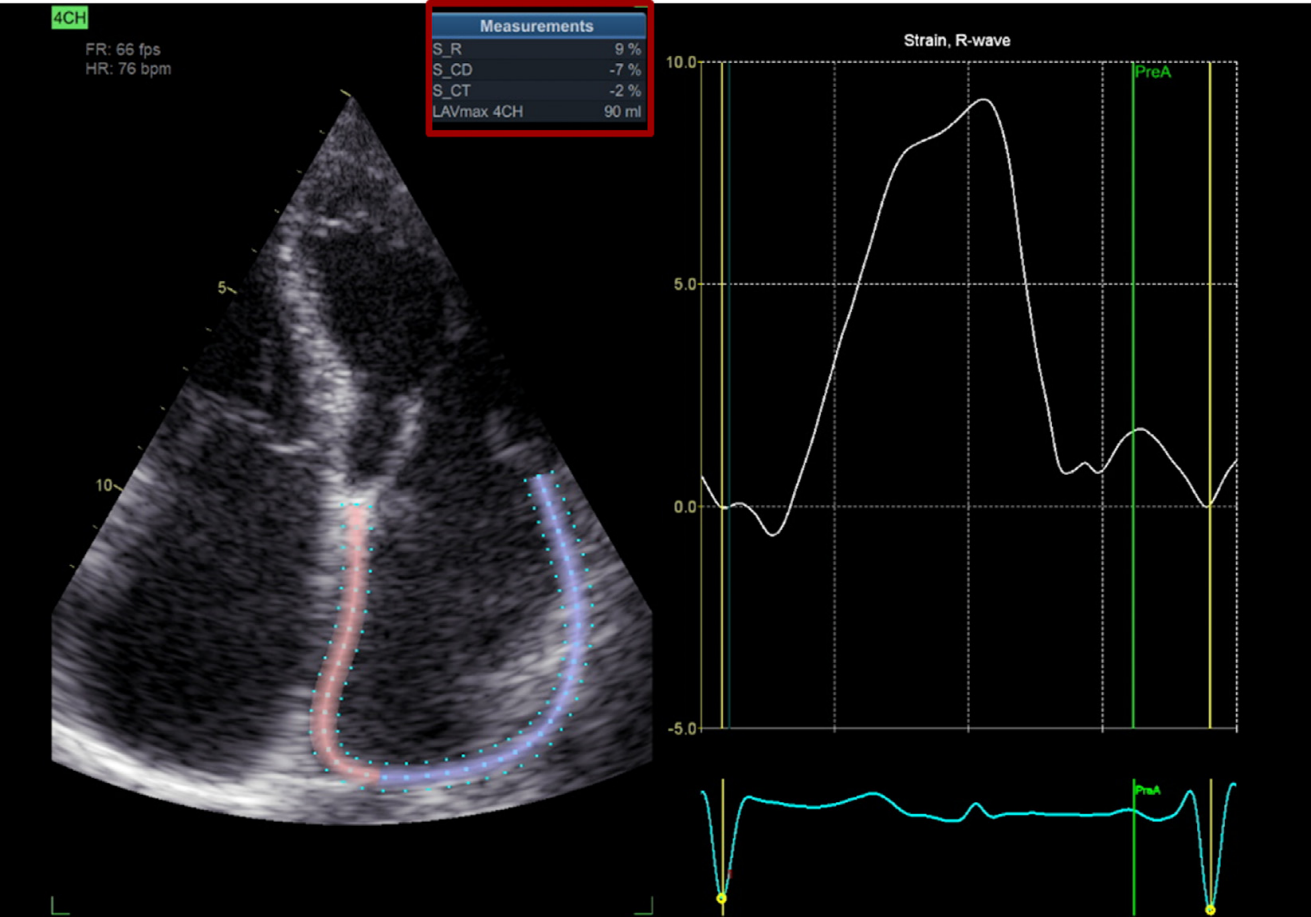
Left Atrial Strain: Significantly Low

Noninvasive assessment of LA fibrosis is challenging. A significant correlation is reported between the lower values of the LA strain and the presence of atrial fibrosis.^6^ The LA strain in our patient is seriously decreased (Figure 3). AF is associated with increased atrial fibrosis, which can explain the presence of interatrial delay in these individuals.^6^ Therefore, we assumed that in our patient, pre-existing atrial fibrosis aggravated the flecainide effect, which is usually much less pronounced.

The unique part of our case is that we were able to detect mechanical atrial dyssynchrony with echocardiography (Video 1).

The final decision for the patient was optimal medical therapy, no antiarrhythmic drugs, and a short follow-up.

## Follow-Up

After 3 months, the patient was in sinus rhythm with a normal P-wave, with persistence of first-degree AV block, and without any clinical symptoms (Figure 1D).

## Conclusions

The presence of completely split P waves on the ECG is a rare and still unclear phenomenon. In our case, long interatrial conduction delay was found in an asymptomatic patient, and it was favored by the use of antiarrhythmic drugs. Careful examination of the surface ECG was used for hypothesis generation in our case and confirmed by the use of echocardiography, which is a safe, accessible, and inexpensive method. Detection of different timings of atrium activation by observing the opening of AV valves and correlating those to the P waves in the ECG is easy and useful. Our case supports the main role that echocardiography can play in the diagnostic process of interatrial delay disorders.

## Funding Support and Author Disclosures

Drs Bertolone and Valeriano are supported by a research grant from the CardioPaTh PhD Program. Dr Oliveira is a part of the CardioPaTh PhD program without grant support. All other authors have reported that they have no relationships relevant to the contents of this paper to disclose.

## Appendix

Video 1 Dyssynchrony of the Opening of the Atrioventricular Valves
